# Supplementary material for: Resolving the full spectrum of human genome variation using Linked-Reads
Source: Genome Res. 2019 Apr;29(4):635–45. doi: 10.1101/gr.234443.118 (PMC6442396; doi:10.1101/gr.234443.118)
Supplement: Supplemental Material [file supp_gr.234443.118_Supplemental_longranger-2.2.zip › longranger-2.2.tar.bz2!/lr2.2-public/lib/stan/cmdstan/stan/doc/api/html/autodiff_8hpp.html]

Stan: src/stan/agrad/autodiff.hpp File Reference


|  |  |
| --- | --- |
|  | Stan  2.5.0  probability, sampling & optimization |


- Main Page
- Related Pages
- Namespaces
- Classes
- Files

- File List
- File Members

All Classes Namespaces Files Functions Variables Typedefs Enumerations Enumerator Friends Macros Pages

- src
- stan
- agrad

Namespaces |
Functions

autodiff.hpp File Reference

`#include <stan/math/matrix/Eigen.hpp>`  
`#include <stan/agrad/rev.hpp>`  
`#include <stan/agrad/fwd.hpp>`

Go to the source code of this file.

|  |  |
| --- | --- |
| Namespaces | |
|  | stan |
|  | Probability, optimization and sampling library. |
|  | |
|  | stan::agrad |
|  | Function gradients via reverse-mode automatic differentiation. |
|  | |

|  |  |
| --- | --- |
| Functions | |
| template<typename T , typename F > | |
| void | stan::agrad::derivative (const F &f, const T &x, T &fx, T &dfx\_dx) |
|  | Return the derivative of the specified univariate function at the specified argument. More... |
|  | |
| template<typename T , typename F > | |
| void | stan::agrad::partial\_derivative (const F &f, const Eigen::Matrix< T, Eigen::Dynamic, 1 > &x, int n, T &fx, T &dfx\_dxn) |
|  | Return the partial derivative of the specified multiivariate function at the specified argument. More... |
|  | |
| template<typename F > | |
| void | stan::agrad::gradient (const F &f, const Eigen::Matrix< double, Eigen::Dynamic, 1 > &x, double &fx, Eigen::Matrix< double, Eigen::Dynamic, 1 > &grad\_fx) |
|  | Calculate the value and the gradient of the specified function at the specified argument. More... |
|  | |
| template<typename T , typename F > | |
| void | stan::agrad::gradient (const F &f, const Eigen::Matrix< T, Eigen::Dynamic, 1 > &x, T &fx, Eigen::Matrix< T, Eigen::Dynamic, 1 > &grad\_fx) |
|  | |
| template<typename F > | |
| void | stan::agrad::jacobian (const F &f, const Eigen::Matrix< double, Eigen::Dynamic, 1 > &x, Eigen::Matrix< double, Eigen::Dynamic, 1 > &fx, Eigen::Matrix< double, Eigen::Dynamic, Eigen::Dynamic > &J) |
|  | |
| template<typename T , typename F > | |
| void | stan::agrad::jacobian (const F &f, const Eigen::Matrix< T, Eigen::Dynamic, 1 > &x, Eigen::Matrix< T, Eigen::Dynamic, 1 > &fx, Eigen::Matrix< T, Eigen::Dynamic, Eigen::Dynamic > &J) |
|  | |
| template<typename F > | |
| void | stan::agrad::hessian (const F &f, const Eigen::Matrix< double, Eigen::Dynamic, 1 > &x, double &fx, Eigen::Matrix< double, Eigen::Dynamic, 1 > &grad, Eigen::Matrix< double, Eigen::Dynamic, Eigen::Dynamic > &H) |
|  | |
| template<typename T , typename F > | |
| void | stan::agrad::hessian (const F &f, const Eigen::Matrix< T, Eigen::Dynamic, 1 > &x, T &fx, Eigen::Matrix< T, Eigen::Dynamic, 1 > &grad, Eigen::Matrix< T, Eigen::Dynamic, Eigen::Dynamic > &H) |
|  | |
| template<typename T1 , typename T2 , typename F > | |
| void | stan::agrad::gradient\_dot\_vector (const F &f, const Eigen::Matrix< T1, Eigen::Dynamic, 1 > &x, const Eigen::Matrix< T2, Eigen::Dynamic, 1 > &v, T1 &fx, T1 &grad\_fx\_dot\_v) |
|  | |
| template<typename F > | |
| void | stan::agrad::hessian\_times\_vector (const F &f, const Eigen::Matrix< double, Eigen::Dynamic, 1 > &x, const Eigen::Matrix< double, Eigen::Dynamic, 1 > &v, double &fx, Eigen::Matrix< double, Eigen::Dynamic, 1 > &Hv) |
|  | |
| template<typename T , typename F > | |
| void | stan::agrad::hessian\_times\_vector (const F &f, const Eigen::Matrix< T, Eigen::Dynamic, 1 > &x, const Eigen::Matrix< T, Eigen::Dynamic, 1 > &v, T &fx, Eigen::Matrix< T, Eigen::Dynamic, 1 > &Hv) |
|  | |
| template<typename F > | |
| void | stan::agrad::grad\_tr\_mat\_times\_hessian (const F &f, const Eigen::Matrix< double, Eigen::Dynamic, 1 > &x, const Eigen::Matrix< double, Eigen::Dynamic, Eigen::Dynamic > &M, Eigen::Matrix< double, Eigen::Dynamic, 1 > &grad\_tr\_MH) |
|  | |


---

[ Stan Home Page ]

*© 2011–2014,
Stan Development Team.*
